# Improved mesophyll–bundle sheath connectivity is achieved via different mechanisms in C_2_ vs C_4_ *Alternanthera*

**DOI:** 10.1111/nph.71218

**Published:** 2026-04-27

**Authors:** Hattie R. Roberts, Roxana Khoshravesh, George Rumble, Tamara Hernández‐Verdeja, Marjorie R. Lundgren

**Affiliations:** ^1^ Lancaster Environment Centre University of Lancaster Bailrigg Lancaster LA1 4YQ UK

**Keywords:** *Alternanthera*, bundle sheath, C_2_ photosynthesis, C_4_ photosynthesis, confocal microscopy, pit fields, plasmodesmata, vein density

## Abstract

- Connectivity between mesophyll (M) and bundle sheath (BS) cells must improve during the evolution of C_4_ photosynthesis to facilitate large metabolite fluxes between these cell types, but the trait combinations that enhance M–BS connectivity and the points at which these enhancements occur along the C_3_ to C_4_ evolutionary trajectory remain unknown.
- We investigated shifts in M–BS connectivity that accompanied photosynthetic diversification within the eudicot genus *Alternanthera* from the agriculturally important Amaranthaceae family. We combined transmission electron microscope and confocal microscopy to reconstruct three‐dimensional internal leaf structures of C_3_, C_2_, and C_4_ *Alternanthera* species to reveal differences in the M–BS interface and cell‐to‐cell communication capacity.
- We found that M–BS connectivity increases stepwise from C_3_ to C_2_ and to C_4_ species, but these shifts are achieved via different combinations of underlying traits in C_2_ vs C_4_ species. Specifically, increases to vein density were unique to C_2_ whilst enhancements to BS cell, pit field, and plasmodesmata densities were unique to C_4_ *Alternanthera* species. Both C_2_ and C_4_ *Alternanthera* shorten BS cell length, which may facilitate tighter BS packing to enhance M–BS connectivity.
- Our findings highlight the diverse mechanisms through which photosynthetic innovation can emerge, providing valuable insight for evolutionary biology and agricultural bioengineering efforts.

## Graphical abstract

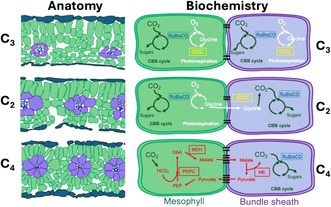

## Introduction

Most plant species, including major crops such as rice, wheat, and soya, utilise only the ancestral C_3_ pathway of photosynthesis. However, maintaining a positive carbon balance can be challenging for C_3_ plants under dry and warm environments, as these conditions induce high rates of photorespiration (Bauwe *et al*., 2010). When the ratio of carbon to oxygen available within the leaf decreases under these environments, oxygenation of the main photosynthetic enzyme Ribulose‐1,5 bisphosphate carboxylase/oxygenase (RuBisCO) generates the toxic compound 2‐phosphoglycolate that must be detoxified through the photorespiration pathway at significant metabolic and energetic costs (Leegood, 2007). Indeed, photorespiration releases roughly a quarter of carbon entering the phosphoglycolate pool, which, along with high energetic costs, significantly limits crop productivity (Betti *et al*., 2016; Walker *et al*., 2016). Carbon concentrating mechanisms (CCMs) have repeatedly evolved in both monocot and eudicot lineages to minimise these photorespiratory costs (R. F. Sage *et al*., 2011; Bräutigam & Gowik, 2016; Gilman *et al*., 2023).

**Fig. 1:**
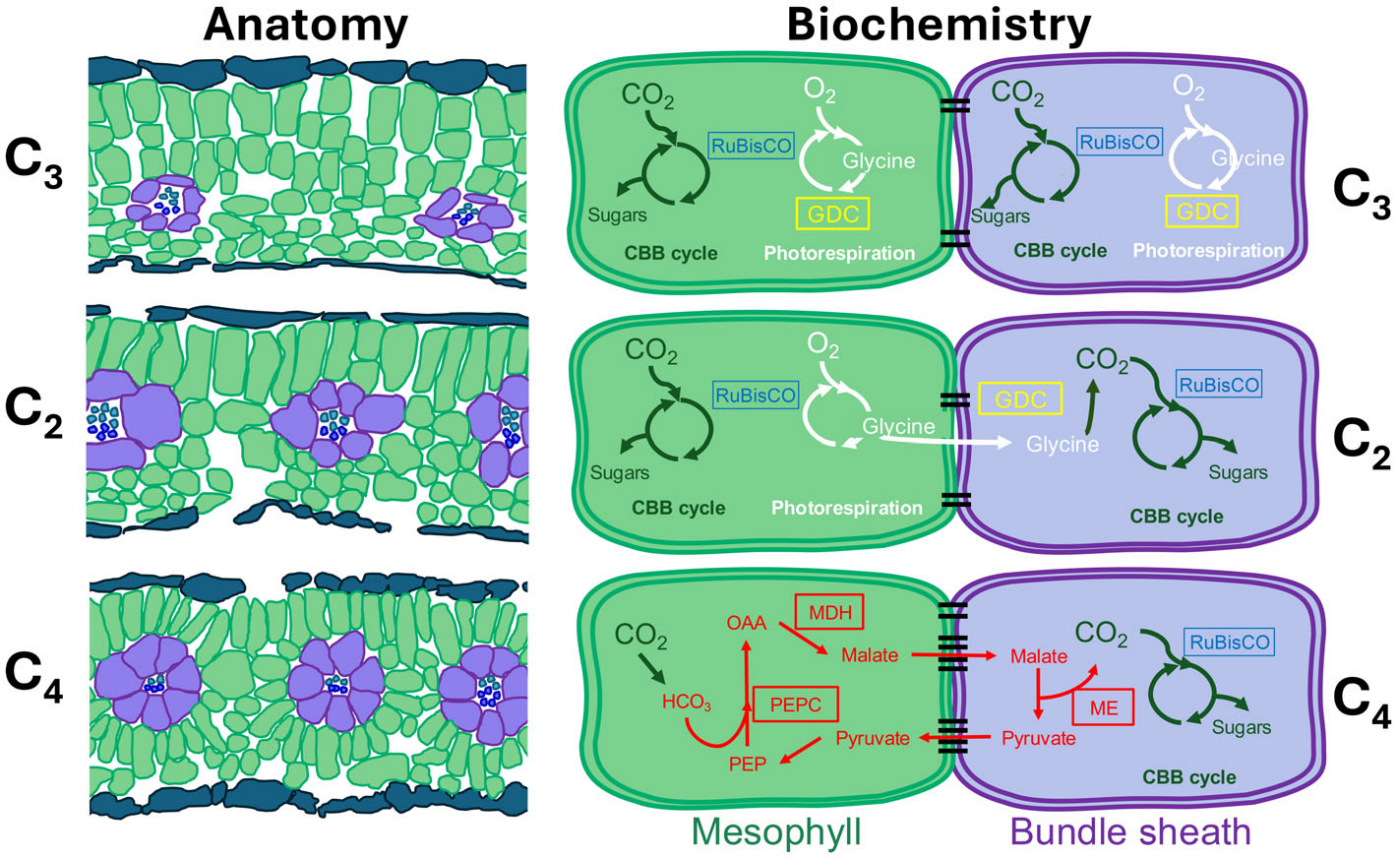
Leaf anatomy and biochemistry of C_3_, C_2_, and C_4_ plants. Anatomically (left), mesophyll (M; green) and bundle sheath (BS; purple) cells form two adjacent compartments, with BS cells surrounding vascular bundles, forming veins. Biochemically (right), organic acids and other metabolites are shuttled via plasmodesmata (black lines adjoining cells). The glycine decarboxylase complex (GDC) is found within M and BS cells of C_3_ plants, but only active within the BS cells of C_2_ plants. Therefore, when photorespiration is initiated in the M of C_2_ plants, the glycine produced accumulates in the M before diffusing (via a concentration gradient) into BS cells, where it can be decarboxylated by GDC. The released carbon concentrates within this BS compartment where it can be reassimilated via Ribulose‐1,5‐bisphosphate carboxylase/oxygenase (RuBisCO) into the Calvin–Benson–Bassham (CBB) cycle. In C_4_ plants, initial fixation of CO_2_ occurs within M cells, catalysed by phosphoenolpyruvate carboxylase (PEPC), which exclusively binds CO_2_. The PEPC reaction produces oxaloacetate (OAA) that is reduced via malate dehydrogenase (MDH) to the four‐carbon acids malate and aspartate before being shuttled to BS cells. Within the BS, the CO_2_‐carrying organic acids are decarboxylated by malic enzyme (ME), increasing the relative concentration of CO_2_. As a result, photorespiration is largely suppressed, which increases net CO_2_ assimilation.

The C_4_ photosynthesis CCM effectively decreases photorespiration whilst enhancing carbon‐, nitrogen‐, and water‐use efficiencies compared with plants using only C_3_ photosynthesis (Chollet & Ogren, 1975; Vogan & Sage, 2011; Sage *et al*., 2012). The repeated emergence of C_4_ phenotypes across diverse plant lineages has created a continuum of C_3_–C_4_ evolutionary intermediate stages (Monson & Moore, 1989; Sage *et al*., 2018; Stata *et al*., 2025), some of which use the globally rare C_2_ CCM (Christin *et al*., 2008; Schlüter & Weber, 2016; Lundgren, 2020). Evolutionary transitions between C_3_, C_2_, and C_4_ types require numerous stage‐specific structural and biochemical modification (Fig. 1; Sage *et al*., 2014; Lundgren, 2020; Leung *et al*., 2024; Alvarenga *et al*., 2025; Stata *et al*., 2025). In particular, the C_3_ to C_2_ transition must initiate, and the C_2_ to C_4_ transition optimise, leaf tissue and cellular architecture to improve connectivity between mesophyll (M) and bundle sheath (BS) cells to facilitate metabolite transfer across these cell types.

Connectivity between M and BS cells can improve via changes to several possible underlying traits, including increasing the M–BS interface and potential for metabolite trafficking between these cell types (Leegood, 2008; McKown & Dengler, 2010; Danila *et al*., 2016). Increasing the amount of interfacing contact between M and BS tissue types creates greater surface area to exchange metabolites between these cell types. Changes to vein density and BS size and shape traits will influence the amount of M–BS interface (Olesen, 1975; Christin *et al*., 2013; Lundgren *et al*., 2014). In both monocot and eudicot lineages, vein density is typically greater in C_4_ plants compared with close C_3_ relatives, often via the proliferation of minor rather than major vein orders (McKown & Dengler, 2009; Lundgren *et al*., 2019). Furthermore, C_4_ plants tend to have greater BS area compared with C_3_ relatives, which can be achieved via a variety of changes to underlying structural traits, including vein density and M and BS cell size and packing (Hattersley, 1984; Dengler *et al*., 1994; Lundgren *et al*., 2014). However, changes to vein and BS traits may occur within C_3_ lineages as anatomical enablers that ultimately facilitate the emergence of C_2_ or C_4_ CCMs (Christin *et al*., 2013; Griffiths *et al*., 2013) or may occur later, within C_4_ lineages, to optimise the C_4_ pathway (Lundgren *et al*., 2019).

Communication across the M–BS cell interface occurs via plasmodesmata channels that connect the cytoplasm of adjacent plant cells and facilitate intercellular trafficking of molecules, such as photosynthetic metabolites (Evert *et al*., 1977; Faulkner *et al*., 2008; Tee & Faulkner, 2024). Plasmodesmata channels often congregate into pit fields, which are areas of thinner plant cell wall with reduced cellulose that facilitate the development of plasmodesmata (Evert *et al*., 1977; Botha *et al*., 1993; Gao *et al*., 2022). Because of their important role in facilitating metabolite exchange across the M–BS interface (Danila *et al*., 2016), plasmodesmata tend to occur in higher densities in C_4_ compared with C_3_ plants (Weiner *et al*., 1988; Danila *et al*., 2016; Khoshravesh *et al*., 2020; Schreier *et al*., 2024). Plasmodesma structure may also influence their ability to traffic metabolites between cells (Burch‐Smith *et al*., 2011), as plasmodesma becomes more complex with development (Oparka *et al*., 1999), and in response to external cues such as flux demands (Alonso‐Cantabrana *et al*., 2018; Gao *et al*., 2022) and light environment (Sowiński *et al*., 2007; Brunkard & Zambryski, 2019).

The extent to which modifications to each underlying trait help to shift the overall connectivity between M and BS cells during the evolutionary transition from C_3_ to C_4_ has yet to be elucidated. Recent research is starting to clarify evolutionary trends in M–BS communication mediated by shifts in plasmodesmata initiation (Danila *et al*., 2016; Schreier *et al*., 2024; Tsang *et al*., 2024; Aleksejeva & Schreier, 2025). However, little is known about the interaction between multiple underlying traits that function together to enhance M–BS connectivity in species using C_2_ and C_4_ CCMs and to what extent C_2_ phenotypes bridge the anatomical gap between C_3_ and C_4_ intercellular connectivity. Understanding the structural changes that distinguish C_3_, C_2_, and C_4_ phenotypes at the leaf intercellular level is critical for C_2_ and C_4_ crop engineering programmes. Here, we use high‐resolution two‐ and three‐dimensional (2D and 3D) reconstructions of internal leaf structure to comprehensively reveal the suites of modifications that function together to increase the M–BS interface and communication that may have accompanied the evolutionary transitions from C_3_ to C_2_ and C_4_ photosynthetic types in *Alternanthera*, a genus from the agriculturally important Amaranthaceae family, for which we have a clearly established evolutionary history of photosynthetic diversity (e.g. Rajendrudu *et al*., 1986; Devi & Raghavendra, 1993; Gowik *et al*., 2006; Sage *et al*., 2007) but completely lack explanation of improved M–BS connectivity across these evolutionary transitions.

## Materials and Methods

### Plant material

We germinated *Alternanthera sessilis* L. (C_3_), *Alternanthera bettzickiana* Regel (C_3_), *Alternanthera tenella* Colla (C_2_), *Alternanthera pungens* Kunth (C_4_ NADP‐ME), and *Alternanthera caracasana* Kunth (C_4_ NADP‐ME) seeds on wet filter paper in Petri dishes for 5 d under glasshouse conditions. We transplanted seedlings onto a 3 : 1 ratio of John Innes No. 2 compost : perlite in 1‐l pots and irrigated plants daily and fertilised weekly with Dyna‐Gro liquid grow plant food 7‐9‐5 (Orchid Accessories Ltd, Oxon, UK). Once plants were established, we moved cuttings from six replicate plants per species into a controlled environment growth chamber (Snijders Labs, Tilburg, the Netherlands) set to 25°C : 20°C, day : night temperatures over a 14‐h photoperiod set to 400 μmol m^−2^ s^−1^ light intensity, 55% relative humidity, and ambient (*c*. 415 ppm) [CO_2_]. After 2 wk in the growth chamber, we sampled tissue from between two and five (depending on plant size and leaf availability) newly mature, fully expanded leaves from five biological replicate plants (*c*. 30 d old) per species within the first 2 h of the photoperiod.

### Optical leaf clearing for confocal microscopy

We optically cleared and stained leaf sections following a protocol modified from Hasegawa *et al*. (2016). When sampling leaf sections, we avoided the edges, tip, base, and midrib of developmentally similar young, fully expanded leaf blades, which allowed us to sample the middle and widest region of the leaf. In doing so, we cut 3–6 mm uneven four‐sided trapezoid leaf fragments, which allowed adaxial and abaxial surfaces to be distinguished. We infiltrated samples in Carnoy's fixative (1 : 4 glacial acetic acid to absolute ethanol) in borosilicate glass vials at room temperature (RT) overnight; replacing with fresh fixative if solutions became saturated. Once the samples had lost all pigments, we placed them in serially decreasing ethanol concentrations of 80%, 70%, 50%, 25%, and RO water for 1 h each at RT. We then replaced RO water with 2% w/v sodium hydroxide (S5881; Sigma Aldrich) until leaf samples were clear, but for no longer than 1 h. After rinsing the samples with RO water three times for 15 min, we used 0.6% fluorescent brightener 28 disodium salt (i.e. calcofluor white, 910090; Sigma Aldrich) in 0.01 M phosphate‐buffered saline (PBS) to stain the cellulose. Samples were refrigerated overnight in the dark to prevent bleaching and then rinsed twice with RO water and twice with 0.01 M PBS. We replaced the sample's solution with 97% 2,2′‐thiodiethanol (TDE, 166782; Sigma Aldrich) in PBS and stored for at least 2 d at 4°C, until the sections appeared transparent. For each biological replicate, we mounted four samples per slide with a 65 μl gene frame (1156024; Fisher Scientific, Loughborough, UK), such that two adaxial and two abaxial sides faced the coverslip. We filled frames with TDE, covered with coverslips, and stored in the dark at 4°C until imaging.

### Confocal microscopy

We used a Zeiss LSM 880 laser scanning confocal microscope (Carl Zeiss Microscopy, Deutschland GmbH) to image veins, individual BS cells, and pit fields (Supporting Information Fig. S1). We detected the fluorescence brightener 28 using 405 nm argon diode laser excitation and a 410–473 nm filter. We visualised veins in three nonoverlapping leaf areas per biological replicate under the 10× objective lens, using a line average of four across a 1024 × 1024 resolution per region of interest (ROI). We adjusted laser power and focal plane for each leaf section to maximise visibility of the veins. We used a 40× oil immersion objective lens to image pit fields and BS cells. We generated Z‐stacks with the following settings: slice interval/axial resolution of 0.5 μm, a pinhole of 1 AU, line averaging of four, lateral resolution of 0.2 μm, and a frame size of 212.55 μm^2^. We generated several Z‐stacks across five replicate plants per species to ensure that we could characterise a total of at least 10 M–BS interfaces along adaxial‐facing BS cells and 10 along abaxial‐facing BS cells (Fig. S2). We used fiji (Schindelin *et al*., 2012) to analyse images and provide quantitative average measurements of traits, as described below. Data are provided in Dataset S1.

### Vein density

To calculate vein density, we imaged veins from three cleared nonoverlapping leaf sections on five replicate plants per species on a Zeiss LSM 880 laser scanning confocal microscope, as described above. We adjusted the laser power and focal plane to maximise the visibility of veins on each leaf section (Fig. S1d). We used the freehand line tool in fiji (Schneider *et al*., 2012) to trace all visible veins within a given area of leaf section along the paradermal plane (i.e. the plane that runs parallel to the epidermis). We quantified total vein length per section then calculated vein density (μm μm^−2^) as the total vein length (μm) per leaf area (μm^2^). We averaged this vein density metric across the three leaf sections per plant to get a single value. We did this for all five biological replicates per species.

### Bundle sheath cell traits

We used the *MorphLibJ* plugin (Legland *et al*., 2016) in fiji to perform morphological segmentation on confocal Z‐stacks that were imaged under a 40× objective lens (Figs S1c, S2). This allowed us to distinguish and characterise individual BS cells. To perform morphological segmentation, we first corrected Z‐stack images for bleaching using the histogram matching function and used the rectangle drawing tool to select only slices that contained BS cells. Next, we selected the segmentation tolerance value that best segmented individual cells for each Z‐stack. We smoothed the cell selections twice to refine the segmentation results by effectively removing noise and smoothing cell boundaries. To select individual cells, we applied label size filtering to filter out segments smaller than cells and manually selected and removed any remaining non‐BS segments. Finally, we calculated the *number of individual BS cells* and *BS cell volume* traits per vein length, using the *Analyse Regions tool in MorphLibJ* (Figs S3, S4). Due to limitations of the light penetration depth within our leaf samples, we quantified BS cell traits from BS cells that were close to the abaxial and adaxial poles (e.g. Fig. S3), rather than lateral BS cells. After confirming that adaxial and abaxial BS cell traits did not significantly differ, we combined data from cells of these two areas for the BS cell traits presented below.

### Per cent bundle sheath periphery interfacing with mesophyll tissue

We calculated the per cent of BS cell periphery that was in direct contact with M tissue (% BS periphery interfacing with M tissue) using 40× confocal Z‐stacks on an average of three leaf sections from each of the five replicate plants per species. To do this, we first reoriented the confocal Z‐stacks horizontally to outline and measure the periphery of BS in the cross‐sectional plane and the length (in μm) of regions of contact between M and BS tissues using the straight‐line drawing tool in fiji. To calculate the % BS periphery interfacing with M tissue, we divided the total length of interfacing M–BS cells by total BS periphery and then multiplied by 100.

### Mesophyll–bundle sheath pit field density

We determined the density of pit fields along the M–BS interface using processed confocal Z‐stacks collected on the Zeiss LSM 880 laser scanning confocal microscope. Using fiji software (Schindelin *et al*., 2012), we identified pit fields as regions of cell wall that fluoresced less via calcofluor white, due to low‐cellulose content (Fig. S4). First, we sub‐sectioned a range of slices that included a single BS‐M interface section and contained visible pit fields. Next, we combined these slices via Z‐projection, selecting SD as the projection type. We then outlined the borders of the interface to generate ROIs, measured the area of the ROI, converted the image to a binary mask, did manual thresholding to eliminate pit fields, and finally measured the area of the thresholded ROI (Fig. S4). The latter measurement represented the area of the interface minus the pit field. We determined the pit field area as the area of the interface before thresholding minus the area after thresholding. Finally, we calculated M–BS pit field density by dividing the pit field area by the total measurement area then multiplying by 100. We did this measurement on 10 M–BS interfaces each from adaxial‐ and abaxial‐facing BS cells, yielding 20 calculations from each replicate plant and a total of 100 interface measurements across the five replicate plants per species.

### Transmission electron microscopy

To quantify plasmodesmata traits along the M–BS interface, we fixed, embedded, and sectioned leaf samples using methods modified from Khoshravesh *et al*. (2017) and Orr *et al*. (2020). Briefly, we sampled tissue from developmentally similar, young and fully expanded leaves, avoiding the base, tip, edges, and midrib of the leaf blade to target the middle and widest section of the leaf. We fixed 2‐ to 3‐mm leaf tissue samples in 2% glutaraldehyde (AGR1012; Agar Scientific), 4% paraformaldehyde (50980487; Fisher Scientific), and 0.01 M PBS overnight. We then washed tissue in 0.01 M PBS three times for 30 min each and replaced the buffer with 2% osmium tetroxide (Agar Scientific) in 0.01 M PBS for a minimum of 2 h or until the tissue was uniformly blackened. We rinsed samples with 0.01 M PBS three times for 30 min each and once with RO water and then dehydrated the samples using an ethanol series of 10% increments increasing from 10 to 100% for 1 h each. We embedded the leaf samples in LR White resin (Agar Scientific) using gelatine capsules (as in Khoshravesh *et al*., 2017) and then sectioned these blocks into ultra‐thin sections (50–70 nm thickness) using a Reichert‐Jung Ultracut E ultramicrotome (Reichert‐Jung; Leica Microsystems, Germany). We collected the ultra‐thin sections on formvar coated nickel 2 mm × 1 mm grids (AGG2980N; Agar Scientific) and then stained them using the nontoxic uranyl acetate substitute UA‐Zero EM stain (Agar Scientific, Rotherham, UK) for 10 min, following Khoshravesh *et al*. (2017). We imaged the ultra‐thin leaf sections with a JEM‐1010 transmission electron microscope (TEM; JEOL Ltd, Tokyo, Japan) using 80 kV voltage.

### Plasmodesmata traits

We used 8000× magnification TEM images to measure the length of the M–BS interface, then used 30 000× magnification to capture the number and visualise the structure of plasmodesmata along the M–BS interface of five M–BS interface regions from each of five biological replicate plants per species, yielding 25 measurements per species. First, we quantified plasmodesmata frequency and density; as the *number of plasmodesmata per M–BS interface* and *number of plasmodesmata per M–BS interface length*, respectively. Then, we characterised plasmodesmata shape complexity as simple (i.e. solitary or twinned) vs branched/complex. Plasmodesmata were counted as individuals where there was no visible connection between two plasmodesma regardless of its complexity, whilst a branched/complex plasmodesma was counted as one when it was not visibly connected to adjacent plasmodesma. TEM data are provided in Dataset S2 and summarised in Dataset S1.

### Statistical analyses

We collated data in Microsoft Excel (see Datasets S1 and S2) and analysed them in R Studio (v.2023.09.0+463; RStudio Team, 2020). After confirming homogeneity of variance and normal data distributions, we performed one‐way ANOVA to test for the effects of species and photosynthetic type on measured traits. We discuss all *P*‐values in the range of 0.05 to 0.1 as marginal and firmly reject null hypotheses when *P* < 0.05. For traits with significant effects from species or photosynthetic type, we perform Tukey HSD *post hoc* tests in base R (R Core Team, 2021). To investigate the association between species or photosynthetic type with plasmodesmata shape complexity, we used a nonparametric Cramér's *V* correlation coefficient for the two categorical variables (i.e. species and photosynthetic type), considering a Cramér's *V* coefficient > 0.5 as a very strong correlation.

## Results

### Mesophyll–bundle sheath interface traits

Connectivity between M and BS cells is a requirement for C_2_ and C_4_ CCM functionality (Schreier *et al*., 2025). To evaluate when enhancements to M–BS connectivity may have occurred during the evolutionary transition from C_3_ to C_4_ in *Alternanthera*, we characterised leaf structural and cellular traits to reveal differences in the amount of BS periphery that interfaces with M tissue across five *Alternanthera* species that use different photosynthetic types (Dataset S1; Table S1).

**Fig. 2:**
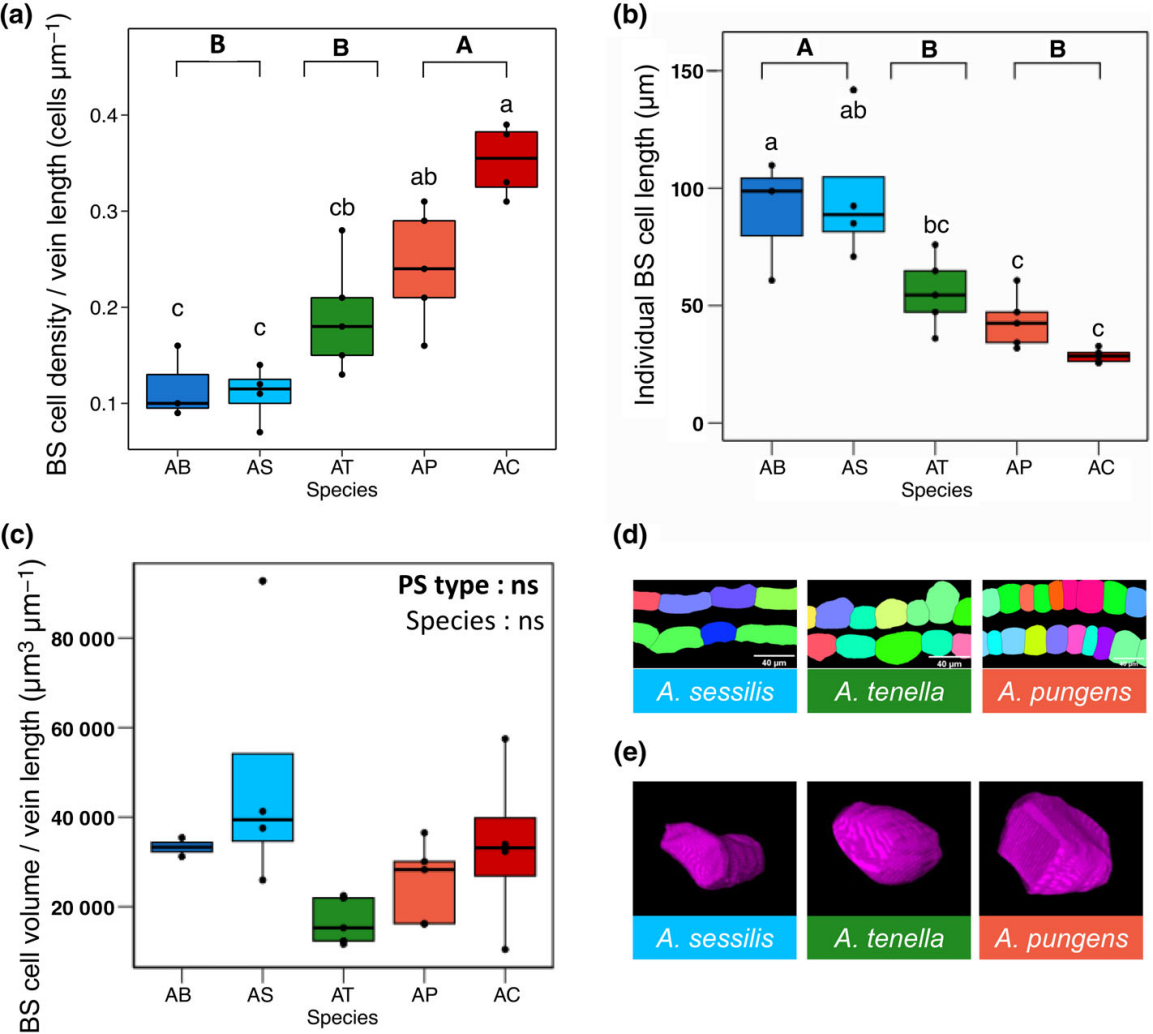
Bundle sheath (BS) cell traits across photosynthetically diverse *Alternanthera* species. For C_3_ (blues) *A. bettzickiana* (AB) and *A. sessilis* (AS), C_2_ (green) *A. tenella* (AT), and C_4_ (reds) *A. pungens* (AP) and *A. caracasana* (AC), we show box plots of (a) BS cell density, showing the number of individual BS cells per fixed vein length; (b) individual BS cell length; and (c) BS cell volume per vein length. Box plots show the median (line within box), interquartile range (box), whiskers (as 1.5× the interquartile range), and any individual outliers (as points). Panels (d) and (e) show representative examples of BS cell density from segmented confocal images of paradermal sections (d; bars, 40 μm) and BS cell volume and shape from three‐dimensional confocal reconstructions of individual BS cells (e) for one C_3_ (*A. sessilis*; blue), C_2_ (*A. tenella*; green), and C_4_ (*A. pungens*; red) species. Compact letter displays represent statistically significant differences between species (letters in regular text directly above boxes) and photosynthetic type (PS type; letters in bold text on top of plots) from one‐way ANOVA *post hoc* Tukey tests (*n* = 3–5 biological replicate plants per species). ns: not significant.

First, we analysed BS cell density using confocal 3D reconstructions of BS cells. We found that BS cells are more tightly packed in C_4_ *Alternanthera* species compared with their C_3_ and C_2_ congeners, such that BS cell density was threefold greater in C_4_ *A. caracasana* than C_3_ *A. sessilis* (Fig. 2a,d). Changes to BS cell density can be achieved via modifications to BS cell size and/or shape. Segmented confocal images of paradermal sections (Fig. 2d) show a decrease in BS cell length along the vein axis in C_2_ and C_4_ species. Indeed, when we analysed BS length, we found that the average individual BS cell is shorter in C_2_ and C_4_, compared with C_3_, *Alternanthera* species (Fig. 2b). However, these shifts to shorter BS cells in C_2_ and C_4_ species are not reflected in BS cell volume/vein length, which did not differ by photosynthetic type or species (Fig. 2c,e). This suggests that BS cells become shorter and wider in C_2_ and C_4_, compared with C_3_ *Alternanthera* species, which would allow them to maintain similar BS volumes despite changes to BS cell shape.

**Fig. 3:**
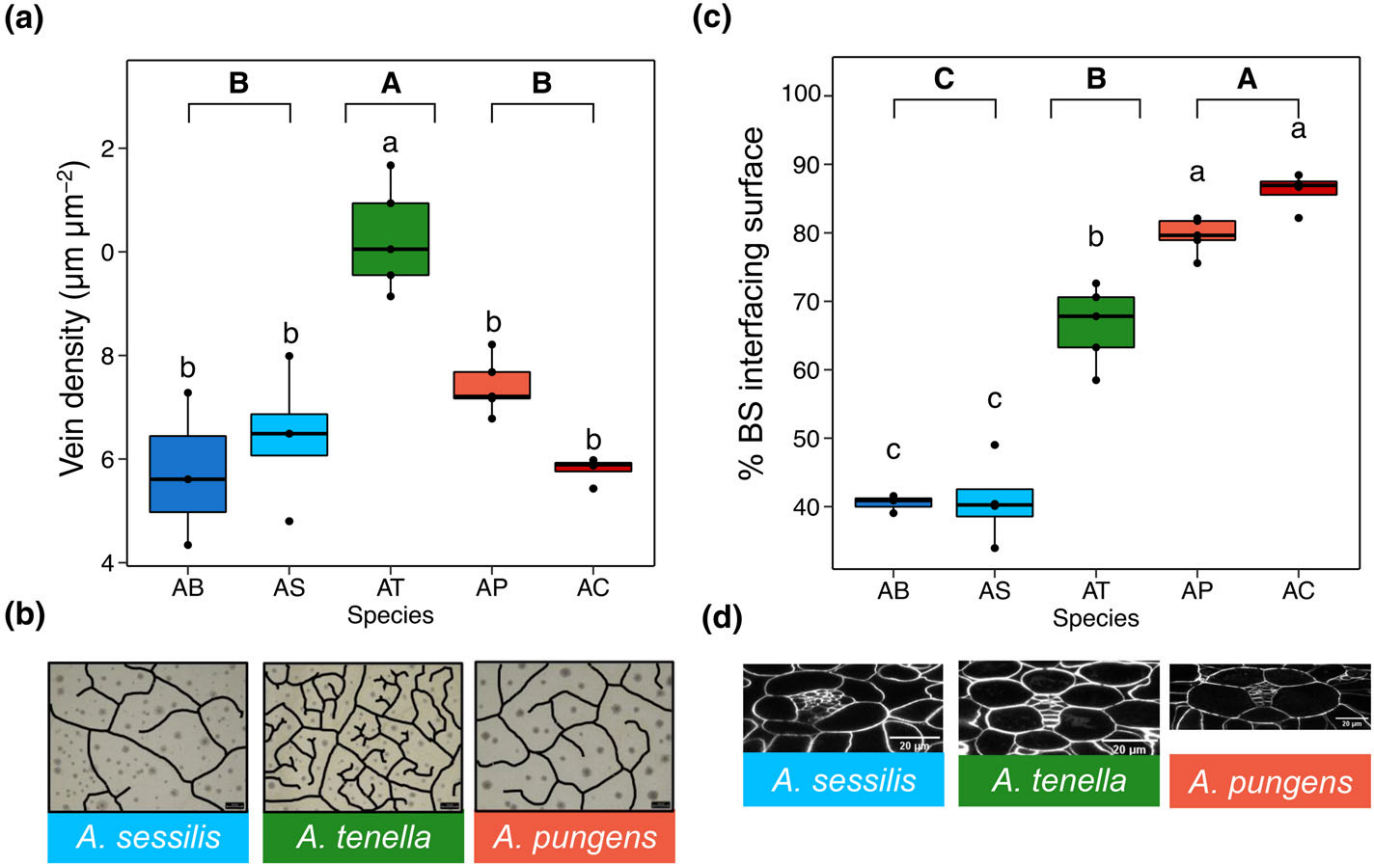
Mesophyll (M)–bundle sheath (BS) interface traits across photosynthetically diverse *Alternanthera* species. For C_3_ (blues) *A. bettzickiana* (AB) and *A. sessilis* (AS), C_2_ (green) *A. tenella* (AT), and C_4_ (reds) *A. pungens* (AP) and *A. caracasana* (AC), we show box plots of vein density (a) and percentage of BS surface area that interfaces with M tissue (c). Box plots show the median (line within box), interquartile range (box), whiskers (as 1.5× the interquartile range), and any individual outliers (as points). Panel b shows representative images of traced lengths of equal area showing veins visible as black lines (bars, 100 μm). Panel (d) shows representative Z‐projected images of the M–BS interface for one C_3_ (*A. sessilis*; blue), C_2_ (*A. tenella*; green), and C_4_ (*A. pungens*; red) species. Compact letter displays represent statistically significant differences between species (letters in regular text directly above boxes) and photosynthetic type (letters in bold text on top of plots) from one‐way ANOVA *post hoc* Tukey tests (*n* = 4–5 replicate plants per species).

Second, we calculated vein density in the paradermal plane of confocal leaf sections as vein length per leaf area. Vein density was significantly higher in C_2_ *A. tenella* (10.3 μm μm^−2^) than all C_3_ (5.7 μm μm^−2^) and C_4_ (6.5 μm μm^−2^) species, such that vein density in C_2_ *A. tenella* was about twice that of both C_3_ *A. bettzickiana* and C_4_ *A. caracasana* (Fig. 3a,b; Table S1). Vein density did not differ between C_3_ and C_4_ *Alternanthera* species, suggesting that incremental increases in vein density to enhance M–BS interface are not associated with C_4_ emergence in this genus. Despite this vein phenotype, the ratio of BS to M tissue area was still greatest in C_4_, smallest in C_3_, and intermediate in C_2_ *Alternanthera* species (Fig. S5).

Last, we analysed the % BS periphery interfacing with M tissue in the cross‐sectional plane of confocal images by calculating the percentage of BS cell wall that was in contact with M cells. Our results reveal that the percentage of BS periphery interfacing with M tissue significantly increases stepwise by photosynthetic type, with C_3_ *Alternanthera* species having 39.4% of their BS surface area touching M cells, C_2_ *A. tenella* having 66.6%, and C_4_ *Alternanthera* species having 82.5% of their BS surface area touching M cells. In other words, the BS of C_4_ species has 20% greater contact with M cells than C_2_ *A. tenella* which, in turn, was *c*. 30% greater than the C_3_ species (Fig. 3c,d; Table S1). This suggests that both C_2_ and C_4_ CCMs in *Alternanthera* may enhance the amount of BS‐M interface – albeit to differing degrees – to potentially facilitate the increased metabolic flux needed for these complex CCMs (Danila *et al*., 2016).

### Mesophyll–bundle sheath communication traits

Metabolic flux between M and BS tissues can potentially be improved via modifications to pit field and plasmodesmata densities and plasmodesma shape complexity (Danila *et al*., 2016; Faulkner, 2018; Tee & Faulkner, 2024; Schreier *et al*., 2025). First, we quantified pit field and plasmodesmata densities along the M–BS interface (Dataset S2). Pit fields were quantified via 3D confocal imagery, whilst plasmodesmata density and complexity were characterised via 2D TEM imagery.

**Fig. 4:**
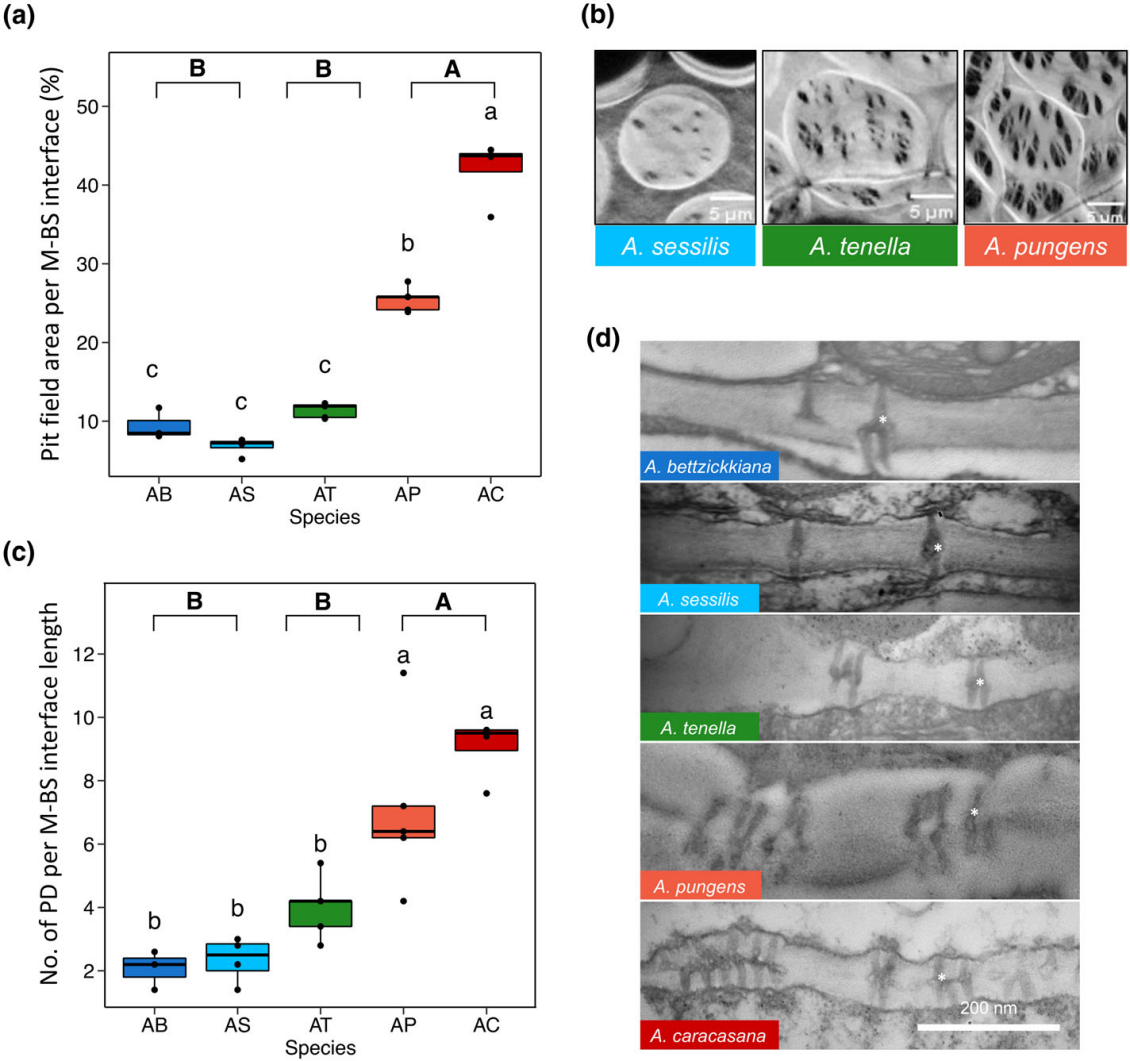
Mesophyll (M)–bundle sheath (BS) communication across photosynthetically diverse *Alternanthera* species. For C_3_ (blues) *A. bettzickiana* (AB) and *A. sessilis* (AS), C_2_ (green) *A. tenella* (AT), and C_4_ (reds) *A. pungens* (AP) and *A. caracasana* (AC), we show box plots of pit field area per M: BS interface (a) and number of plasmodesmata (PD) per M–BS interface length (c). Box plots show the median (line within box), interquartile range (box), whiskers (as 1.5× the interquartile range), and any individual outliers (as points). Panel (b) shows representative cellulose‐stained confocal images of pit fields at the M–BS interface for one C_3_ (*A. sessilis*; blue), C_2_ (*A. tenella*; green), and C_4_ (*A. pungens*; red) species. Bars, 5 μm. Panel (d) shows representative transmission electron microscope (TEM) images of PD (e.g. PD are denoted with white asterisks). Compact letter displays represent statistically significant differences between species (letters in regular text directly above boxes) and photosynthetic type (letters in bold text on top of plots) from one‐way ANOVA and *post hoc* Tukey tests at the *P* < 0.05 level (*n* = 5 biological replicate plants per species).

Pit fields and plasmodesmata were observed along the M–BS interface in all study species (Fig. 3a–c). Pit field area (i.e. pit field area per M–BS interface length) and plasmodesmata frequency and density (i.e. number of plasmodesmata per M–BS interface and per M–BS length, respectively) differed by species and photosynthetic type (Fig. 4; Table S2). Pit field area along the M–BS interface was greater in C_4_ *Alternanthera* species (41.5% in *A. caracasana* and 25.5% in *A. pungens*) than C_2_ (11.4%) and C_3_ (6.8% in *A. sessilis* and 9.5% in *A. bettzickiana*) congeners (Fig. 4a; Dataset S1). Plasmodesmata were more abundant along the M–BS interface of C_4_ (*c*. 8 plasmodesmata μm^−1^) compared with C_2_ (*c*. 4 μm^−1^) and C_3_ (*c*. 2 μm^−1^) *Alternanthera* species (Fig. 4c,d). Plasmodesmata per M–BS interface length mirrored this trend, being greater in C_4_ *Alternanthera* species (0.650 μm^−1^ in *A. caracasana* and 0.853 μm^−1^ in *A. pungens*) than C_2_ (0.278 μm^−1^) and C_3_ (0.218 μm^−1^ in *A. sessilis* and 0.286 μm^−1^ in *A. bettzickiana*) congeners (Table S2). These results suggest that changes to pit fields and plasmodesmata were unique to C_4_ *Alternanthera* species and not apparently required for C_2_ CCM functionality.

**Fig. 5:**
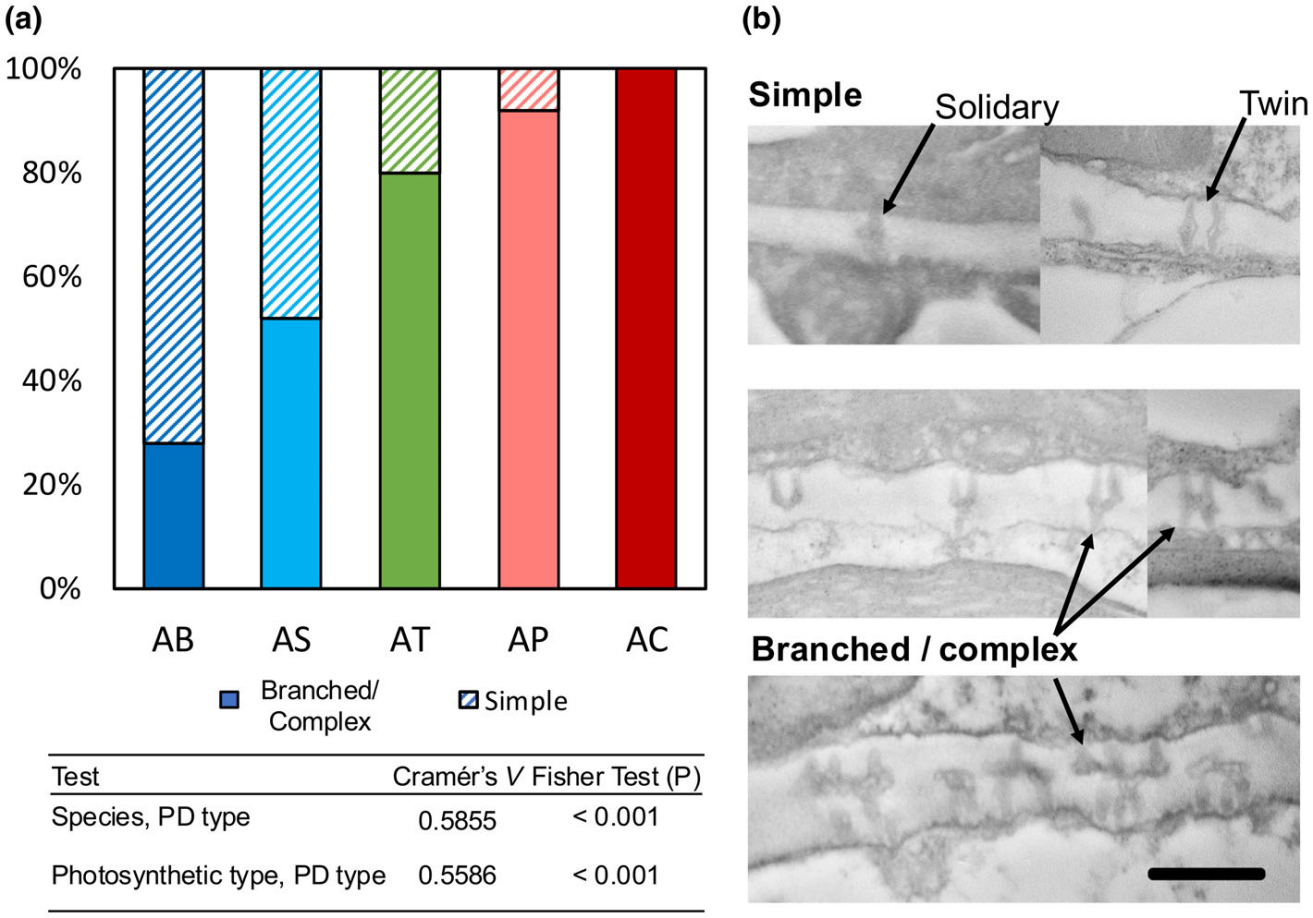
Plasmodesma (PD) shape complexity across photosynthetically diverse *Alternanthera* species. Panel (a) summarises the percentage of PD shape complexity types (simple vs branched/complex) for C_3_ (blues) *A. bettzickiana* (AB) and *A. sessilis* (AS), C_2_ (green) *A. tenella* (AT), and C_4_ (reds) *A. pungens* (AP) and *A. caracasana* (AC). Cramér's *V* coefficient and Fischer test *P*‐value statistics are provided for the effects of species and photosynthetic type. Panel (b) shows representative transmission electron microscope (TEM) images showing example simple (including single and twin PD types; top) and branched/complex (middle and bottom images), as denoted by arrows. Bars, 500 nm.

Imaging at high magnification (30 000×) on the TEM allowed us to characterise plasmodesma structure in detail and, in doing so, we identified a remarkable diversity of plasmodesma structures across the *Alternanthera* species in our study despite sampling leaves at similar developmental stages and regions of the leaf blade. Plasmodesma presented as simple (i.e. solitary or twined) and with a variety of branched and complex structures, including H‐, V‐, Y shapes (Fig. 5b; Dataset S2). We found that plasmodesmata shape complexity increased stepwise by photosynthetic type (Fig. 5a). Simple plasmodesmata were most common in C_3_ species (72% of *A. bettzickiana* and 48% of *A. sessilis*), with fewer simple plasmodesmata in C_2_ *A. tenella* (20%) and fewer still in the C_4_ species (8% in *A. pungens*; simple plasmodesma were not observed in *A. caracasana*). By contrast, the per cent of branched/complex plasmodesmata increased from C_3_ (28% in C_3_ *A. bettzickiana*; 52% in *A. sessilis*) to C_2_ *A. tenella* (80%) and to C_4_ (92% and 100% in C_4_ *A. pungens* and *A. caracasana*, respectively; Dataset S2). This shift in plasmodesmata complexity across photosynthetic types, supported by a Cramér's *V* coefficient of 0.5586 and *P* < 0.001 (Fig. 5a), highlights a possible association between plasmodesmata structural complexity and photosynthetic type.

## Discussion

### Reorientation of BS cell axis may increase M–BS connectivity in both C_2_ and C_4_ *Alternanthera*

We found that BS cells became more densely packed in C_4_ compared with C_2_ and C_3_ *Alternanthera* species. Leaf structure and cellular shape are driven by coordination of complex interactions between transcriptional regulators that establish and maintain 3D developmental axes (Dengler & Nelson, 1999; Satterlee & Scanlon, 2019; Claßen‐Bockhoff *et al*., 2021). Thus, the shift that we observed in BS cell packing was likely mediated by a reorientation of the BS cellular developmental axis that resulted in wider and shorter cells. We did not find that BS cell volume was greater in C_4_ *Alternanthera* species. This result contrasts with previous studies concluding that C_2_ and C_4_ leaves require larger BS cell volumes to house the greater number and/or area of BS organelles needed for C_2_ and C_4_ photosynthesis, as well as to accommodate the large vacuoles that assist in concentrating CO_2_ within BS cells by resisting CO_2_ efflux (Caemmerer & Furbank, 2003; Christin *et al*., 2013). However, a recent study found that BS cell volumes are comparable between C_3_ and C_4_ species from multiple grass lineages (Danila *et al*., 2018). For example, in the grass subtribe *Neurachninae*, which uses the mestome sheath for the BS Calvin–Benson–Bassham cycle, mestome sheath cell volumes did not differ between C_3_, C_2_, and C_4_ species. Instead, wider and shorter cells present an evolutionary shift in mestome sheath cell division within these monocots, enabling an increased mestome sheath cell number and mestome sheath tissue volume per vein length (Khoshravesh *et al*., 2020). Additionally, no correlation was found between the volume of monocot mestome sheath and their CO_2_ compensation point, a trait that effectively distinguishes photosynthetic type (Tolbert *et al*., 1995).

Our finding that BS cell density was greater in C_4_ compared with C_2_ and C_3_, *Alternanthera* implies that evolution of the C_4_ CCM in this genus may be promoted – at least in part – by reducing the length of BS cells axis parallel to veins, allowing more – but shorter – BS cells to be packed along a vein length. These findings not only agree with observations of shorter BS cells in C_4_ grasses, as described above (Danila *et al*., 2018; Khoshravesh *et al*., 2020), but also in C_4_ eudicot *Atriplex* species (Sultmanis, 2018). Furthermore, a review of paradermal images of leaves in the literature indicates that short but numerous BS cells in C_2_, C_4_‐like, and C_4_ species, relative to C_3_ relatives, could be a general trait in the eudicot lineages of *Flaveria* (McKown & Dengler, 2007), *Gynandropsis gynandra* (Huang *et al*., 2017), and *Moricandia arvensis* (Rawsthorne *et al*., 1988). A commonality across these studies, and our own, is that the reduction in BS cell length is accompanied by an increase in the BS cells axis perpendicular to the vein, which results in BS (or mestome sheath) cells to appear larger than C_3_ relatives in the 2D cross‐sectional view (Fig. 3d), whilst BS volume remains consistent across photosynthetic types (Fig. 2c). Our results suggest that *Alternanthera* C_2_ and C_4_ lineages experienced shifts BS shape that resulted in shorter BS cells that can pack more tightly (and thus, increase connecting surface area) compared with C_3_ relatives. Thus, BS cellular shape might be a better predictor of C_2_ and C_4_ function in *Alternanthera* than BS area or volume traits alone.

### Enhanced vein density is not required for C_4_ photosynthesis in *Alternanthera*

We found that vein density was significantly higher in C_2_ *A. tenella* compared with C_3_ and C_4_ congeners. Increases to vein density are often cited as a preconditioning stage of photosynthetic evolutionary transitions, preceding the establishment of the two distinct M and BS photosynthetically relevant compartments, and enabling the evolution of C_2_ and C_4_ CCMs (Muhaidat *et al*., 2011; Lundgren *et al*., 2019). However, the importance of further increases in vein density during the transitions to a C_2_ or C_4_ phenotype varies between closely related species (McKown & Dengler, 2009) and even within species (Lundgren *et al*., 2019). For instance, the only common trait distinguishing C_4_ from non‐C_4_ phenotypes in the photosynthetically diverse monocot grass species *Alloteropsis semialata* is the presence of frequent and regular minor veins, which function to increase overall vein density and BS tissue volume, whilst decreasing M volume (Lundgren *et al*., 2019). However, we also found that the overall ratio of BS to M tissue was higher in C_4_ species compared with C_2_ and C_3_ species, regardless of this vein density pattern. Thus, our results suggest that high vein density was not required to increase the BS : M area ratio or subsequently enhance M–BS connectivity in C_4_ *Alternanthera* species. This differs from reports in the literature where vein density was found to increase stepwise from C_3_ to C_2_ and to C_4_ congeners in the eudicot genera *Euphorbia* (T. L. Sage *et al*., 2011) and *Flaveria* (McKown & Dengler, 2007). However, studies in the genus *Heliotropium* found that C_3_, C_2_, and C_4_ congeners presented with similar vein density (Muhaidat *et al*., 2007, 2011). These disparate trends in vein density along the photosynthetic continuum across diverse plant lineages suggest that vein density may not necessarily contribute directly to C_2_ and C_4_ function and, instead, may be just one of many traits that can enhance M–BS connectivity.

### Enlarged M–BS interface may enhance M–BS connectivity in both C_2_ and C_4_ *Alternanthera*

We found that the M–BS interface is larger in C_2_ and C_4_ *Alternanthera* species compared with their C_3_ congeners. This result is consistent with previous studies in monocots that recorded a five times greater M–BS interface in C_4_ species (*Setaria viridis*, *Zea mays*) compared with their C_3_ counterparts (*Oryza sativa, Triticum aestivum*; Danila *et al*., 2016, 2018). Within our study, increases to the M–BS interface between C_3_ and C_4_ *Alternanthera* species may also reflect reductions in the size of air spaces between adjacent M cells, as the strength of the metabolite transport between M and BS cells increases (see Fig. 1 for representative air space examples), which would be consistent with previously reported, yet unquantified, observations that M cells in C_4_ *Flaveria* species (*F. bidentis* and *F. trinervia*) have greater overall contact with BS cells than C_3_ or protokranz congeners (*F. pringlei* and *F. robusta*; Kümpers *et al*., 2017).

The stepwise increases in M–BS connectivity that we identified between C_3_, C_2_, and C_4_ species align with previous studies showing that metabolite diffusion or active transport into the BS increases from C_3_ to C_2_, and again from C_2_ to C_4_ photosynthetic types to accommodate the progressively enhanced photosynthetic activity in the BS (Keeley & Rundel, 2003; Bräutigam & Gowik, 2016). However, the timing of each underlying trait acquisition along the C_3_ to C_4_ evolutionary continuum may be lineage specific. We can postulate that the transition from C_3_ to C_2_ photosynthesis in *Alternanthera* was accompanied by increases to vein density, BS cell shape, and the % BS periphery interfacing with M tissue, which may function together to increase M–BS connectivity. Together these enhancements to M–BS connectivity likely facilitated the C_2_ CCM to function via improved potential for metabolite transfer between these cell types. Enhancements to BS cell shape and % BS periphery interfacing with M tissue may have been co‐opted and enhanced further for C_4_ CCM functionality, whilst modifications to vein density were perhaps either not possible or not required for the C_4_ CCM in *Alternanthera*.

### Enhanced plasmodesmata density is not required for C_2_ photosynthesis in *Alternanthera*

We found that C_4_ *Alternanthera* has greater pit field area and plasmodesmata density, compared with C_2_ and C_3_ congeners. Our findings highlight an important role of plasmodesmata in facilitating the greater M–BS communication needed for the C_4_ pathway, as has previously been reported in both grasses (Danila *et al*., 2016) and eudicots (Schreier *et al*., 2024). We did not, however, identify a shift in pit field area or plasmodesmata density in C_2_ compared with C_3_ *Alternanthera*, which suggests that these cell‐to‐cell communication traits may not be critical for the functionality of the C_2_ glycine shuttle in this eudicot lineage. This contrasts with previous studies that found plasmodesmata density to increase along the evolutionary continuum from C_3_ to C_2_ and again to C_4_ types in the grass subtribe Neurachninae (Khoshravesh *et al*., 2020) and the eudicot *Flaveria* genus (Aleksejeva & Schreier, 2025), which suggests a potential role of the C_2_ phenotype in enhancing M–BS communication in C_4_ evolution in these lineages.

Plasmodesma shape complexity, which increased stepwise in from C_3_ to C_2_ and again C_4_ *Alternanthera* species in our study, might also facilitate enhanced M–BS communication required for the M–BS glycine shuttle and C_4_ pathway in this genus. Plasmodesma shape complexity can define the direction of flux, metabolite size, and rate of transport between cell types, with more complex shapes creating more tightly controlled trafficking and more funnel shaped or unevenly branched forms (i.e. Y shape) effectively driving molecular flux in a single direction (Tee & Faulkner, 2024). If this was the case, then the evolutionary intermediate C_2_ stage may initiate, and the C_4_ stage further improve, an important phenotypic step in defining M–BS metabolic direction and flux via plasmodesma shape complexity. However, plasmodesmata shape complexity does not always enhance cell‐to‐cell communication and has, in fact, been associate with reduced trafficking in some species (e.g. Oparka *et al*., 1999; Roberts *et al*., 2001; Nicolas *et al*., 2017). Thus, potential linkages between plasmodesmata complexity and metabolite trafficking capacity should be directly tested to determine any potential roles that it may play in C_2_ and C_4_ CCM emergence in *Alternanthera*.

### Conclusion

Our study quantified the underlying traits that drive enhanced M–BS connectivity across photosynthetic types in *Alternanthera*. We demonstrate that increases to diverse traits contributed to overall enhancements in M–BS interface and communication in C_2_ and C_4_ species in this eudicot genus. We identified four traits that may enhanced M–BS connectivity in C_2_, compared with C_3_, *Alternanthera* species: increased vein density, shortened BS cell length, higher BS interfacing with M tissue, and enhanced plasmodesmata complexity along the M–BS cellular junctions. By contrast, we found that M–BS connectivity may have been enhanced in C_4_ *Alternanthera*, compared with C_3_ and/or C_2_ congeners via increased BS cell density, larger BS area interfacing with M tissue, greater pit field area, plasmodesmata density, and enhanced plasmodesmata complexity along the M–BS cellular junctions. For most traits, C_2_ *A. tenella* did not present with an intermediate phenotype, as only BS area interfacing with M tissue fell intermediate between the C_3_ and C_4_ congeners. We conclude that BS cell length and initial changes BS area interfacing with M tissue and plasmodesma shape complexity occurred before enhancements to BS cell density, pit field area, and plasmodesmata density during the evolutionary transition from C_3_ to C_4_ in *Alternanthera*. Changes BS area interfacing with M tissue and plasmodesma shape complexity further shifted with the emergence of the C_4_ CCM in this lineage. The high vein density that we observed in C_2_ *Alternanthera* may have arisen after C_2_ emergence in this lineage, to optimise the C_2_ CCM, instead of within the common ancestor of both C_2_ and C_4_ *Alternanthera* lineages. Overall, the enhancements to M–BS interface and communication parameters that we report here may increase transport capacity of CO_2_‐carrying organic acids into the BS and, as such, warrant further investigation. Overall, this study indicates that evolutionary transitions from C_3_ to C_2_ and C_2_ to C_4_ phenotypes are structurally heterogeneous, with these transitions involving diverse combinations of anatomical alterations. Moreover, the order and nature of these acquired anatomical traits likely vary considerably between plant lineages. Improved understanding of the leaf structural shifts that accompanied the repeated emergences of C_2_ and C_4_ phenotypes will facilitate taxa‐specific engineering of these pathways into C_3_ crops to ultimately aid food security.

## Competing interests

None declared.

## Author contributions

RK and MRL designed the study and led the project. GR and RK performed the experiments, with the help of TH‐V. HRR, RK, GR, TH‐V and MRL interpreted the results and wrote the paper.

## Disclaimer

The New Phytologist Foundation remains neutral with regard to jurisdictional claims in maps and in any institutional affiliations.

## Supporting information


**Dataset S1** Mean mesophyll (M)–bundle sheath (BS) interfacing and communication data for each replicate plant.
**Dataset S2** Mean plasmodesmata density and shape complexity along the mesophyll–bundle sheath interface data collected across five replicate plants per each of five research species.

**Fig. S1** Summary of confocal imaging approaches used in this study.
**Fig. S2** Representative 3D reconstruction of bundle sheaths.
**Fig. S3** Representative confocal images of abaxial and adaxial mesophyll and bundle sheath cells.
**Fig. S4** Methodology used to determine pit field percentage per M–BS interface.
**Fig. S5** Comparison between bundle sheath and mesophyll tissue areas.
**Table S1** Nested one‐way ANOVAs for the mesophyll (M)–bundle sheath (BS) interface parameters tested against photosynthetic (PS) type and species.
**Table S2** Nested one‐way ANOVAs for the mesophyll (M)–bundle sheath (BS) communication parameters tested against photosynthetic (PS) type and species.
Please note: Wiley is not responsible for the content or functionality of any Supporting Information supplied by the authors. Any queries (other than missing material) should be directed to the *New Phytologist* Central Office.

## Data Availability

All primary data used to support the findings of this study are provided in Datasets S1 and S2.
