# Supplementary material for: Improved mesophyll–bundle sheath connectivity is achieved via different mechanisms in C2 vs C4 Alternanthera
Source: New Phytol. 2026 Apr 27;250(6):3675–86. doi: 10.1111/nph.71218 (PMC13193408; doi:10.1111/nph.71218)
Supplement: Supplementary file 2 — Fig. S1 Summary of confocal imaging approaches used in this study. Fig. S2 Representative 3D reconstruction of bundle sheaths. Fig. S3 Representative confocal images of abaxial and adaxial mesophyll and bundle sheath cells. Fig. S4 Methodology used to determine pit field percentage per M–BS interface. Fig. S5 Comparison between bundle sheath and mesophyll tissue areas. Table S1 Nested one‐way ANOVAs for the mesophyll (M)–bundle sheath (BS) interface parameters tested against photosynthetic (PS) type and species. Table S2 Nested one‐way ANOVAs for the mesophyll (M)–bundle sheath (BS) communication parameters tested against photosynthetic (PS) type and species. Please note: Wiley is not responsible for the content or functionality of any Supporting Information supplied by the authors. Any queries (other than missing material) should be directed to the New Phytologist Central Office. [file NPH-250-3675-s001.pdf]

New Phytologist Supporting Information

Article title: Improved mesophyll-bundle sheath connectivity is achieved via different underlying mechanisms in C_2 versus C_4 *Alternanthera species*

Authors: Hattie R. Roberts, Roxana Khoshravesh, George Rumble, Tamara Hernández-Verdeja, Marjorie R. Lundgren

Article acceptance date: 24 March 2026

DATASETS

Dataset S1. Mean mesophyll (M)-bundle sheath (BS) interfacing and communication data for each replicate plant. Vein density, bundle sheath cell density and volume, M-BS interfacing surface area, pit field density, and plasmodesmata (PD) density per M-BS interface are presented for the five replicate plants per each of five research species. Details provided in the READ ME tab.

Dataset S2. Mean plasmodesmata (PD) length and shape complexity along the mesophyll-bundle sheath interface across five replicate plants per each of five research species. Details provided in the READ ME tab.

TABLES

Table S1. Nested one-way ANOVAs for the mesophyll (M) – bundle sheath (BS) interface parameters tested against photosynthetic (PS) type and species, nested within photosynthetic type. We consider *p*-values in 0.05 to 0.1 range as marginal (in italics) and firmly reject the null hypotheses when $p < 0.05$ (in bold). The *PS Type* categories are C_3, C_2, and C_4; the *Species* categories include *Alternanthera bettzickiana*, *Alternanthera sessilis*, *Alternanthera tenella*, *Alternanthera pungens* and *A. caracasana*. df, degrees of freedom.

M-BS Interface Parameters	Effect	df	*F*	*P*
BS cell density	PS type	2	31.72	**1.83E-06**
	PS: Species	4	6.71	**0.00712**
Individual BS cell length	PS type	2	21.02	**1.53e-05**
	PS: Species	4	10.95	**1.41E-04**
BS cell volume/vein length	PS type	2	2.285	0.129
	PS: Species	4	1.675	0.202
Vein density	PS type	2	30.02	**9.48E-07**
	PS: Species	4	3.24	*0.0603*
% Interfacing M-BS area	PS type	2	184.55	**1.58E-06**
	PS: Species	4	2.54	**6.94E-11**

Table S2. Nested one-way ANOVAs for the mesophyll (M) – bundle sheath (BS) communication parameters tested against photosynthetic (PS) type and species, nested within photosynthetic type. PD, plasmodesmata. We consider *p*-values in 0.05 to 0.1 range as marginal (in italics) and firmly reject the null hypotheses when $p < 0.05$ (in bold). The *PS Type* categories are C_3, C_2, and C_4; the *Species* categories include *Alternanthera bettzickiana*, *Alternanthera sessilis*, *Alternanthera tenella*, *Alternanthera pungens* and *A. caracasana*. df, degrees of freedom.

**M-BS Communication Parameters**	**Effect**	**df**	***F***	***P***
Pit field area per M-BS interface	PS type	2	388.66	**3.87E-16**
	PS: Species	4	74.97	**9.66E-10**
PD number per M-BS interface	PS type	2	29.52	**3.27E-08**
	PS: Species	4	1.89	0.100
PD number per M-BS interface length	PS type	2	39.62	**5.53e-14**
	PS: Species	4	22.18	**1e-13**

FIGURES

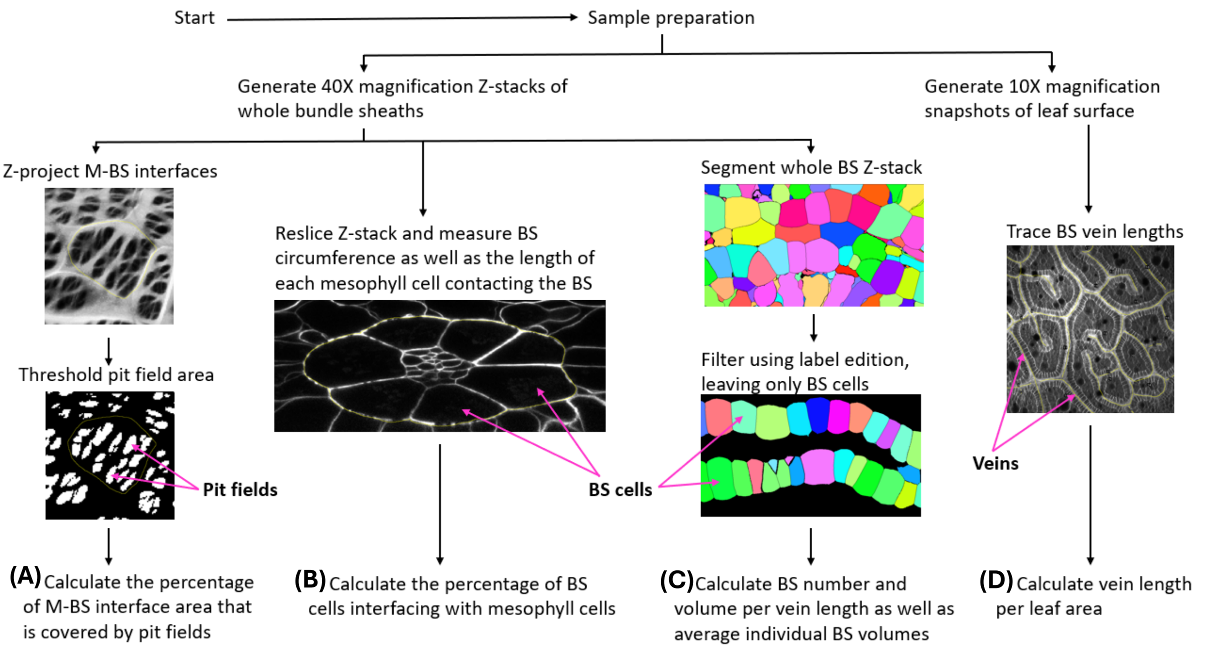

Figure S1. Summary of confocal imaging approaches used in this study. (A) Pit field area was calculated by Z-projecting the slices within a mesophyll (M) – bundle sheath (BS) interface that contained pit fields, visible as the black dots in the top image and white dots in the bottom image. (B) Whole BS Z-stacks were resliced to visualise the M-BS interfaces at multiple points along the BS length and calculate the proportion of the M-BS surface that contacts mesophyll cells. (C) Measurement of whole BS volume and number as well individual BS volumes used the MorphoLibJ plugin to segment the BS Z-stacks and subsequently filter them, leaving only BS cells. Individual BS volumes were measured by further filtering to remove BS cells that were cut by the edge of the image frame. (D) Veins were imaged at low magnification to provide a large area over which vein lengths could be traced and measured. All steps represented by images from the C_4 species *Alternanthera pungens*. M-BS = Mesophyll-Bundle sheath.

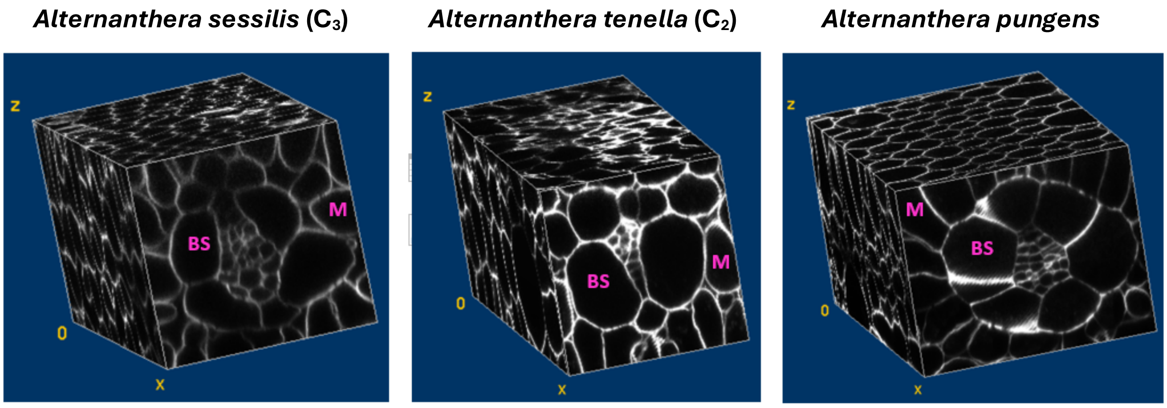

Figure S2. Representative 3D reconstructions of bundle sheaths. *Alternanthera sessilis* (C_3), *A. tenella* (C_2), and *A. pungens* (C_4) bundle sheath 3D reconstructions are shown. Z-stacks were generated from prepared leaf sections stained with fluorescent brightener (*i.e.,* calcofluor white) then imaged using a Zeiss LSM 880 confocal microscope (Carl Zeiss Microscopy Ltd, Deutschland GmbH). 3D volumes were then recreated using the volume viewer plugin within FIJI image analysis software version 2.14.0 (Schindelin *et al.*, 2012). M = mesophyll, BS = bundle sheath.

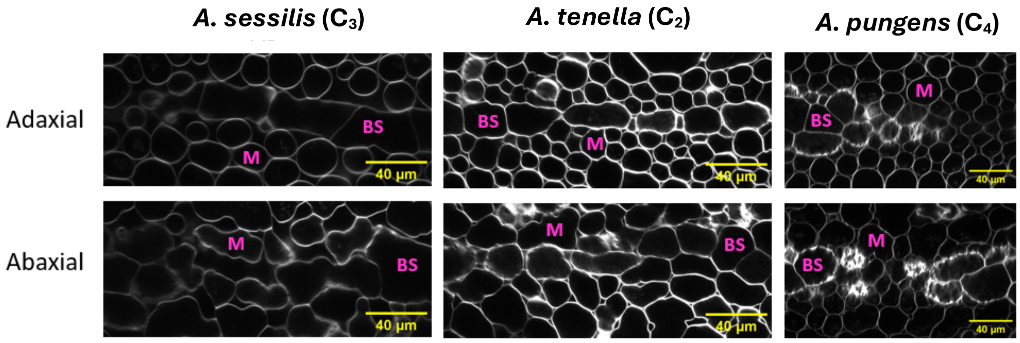

Figure S3. Representative confocal images of abaxial and adaxial mesophyll and bundle sheath cells. Example images of *Alternanthera sessilis* (C_3), *Alternanthera tenella* (C_2), and *Alternanthera pungens* (C_4) were modified using ImageJ software. Cell types are denoted by an M for Mesophyll and BS for Bundle Sheath. Scale is 40 μm.

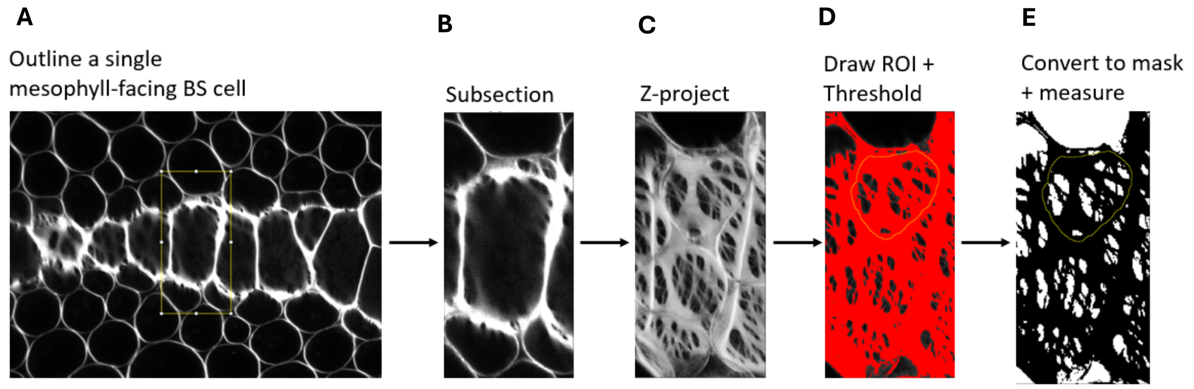

Figure S4. **Methodology used to determine pit field percentage per mesophyll – bundle sheath interface.** Steps included (A) isolating all sections that contained pit fields from (B) one mesophyll (M)-facing bundle sheath (BS) cell, then combining the slices via Z-projection (C) to recreate a 3D reconstruction of the M-BS interface. Next, a region of interest (ROI) was drawn around one M-BS interface (D) before thresholding to generate a binary mask (E). This enabled measurement of the ROI area minus pit fields and subsequent calculation of pit field percentage per M-BS interface. M = mesophyll, BS = bundle sheath, ROI = region of interest.

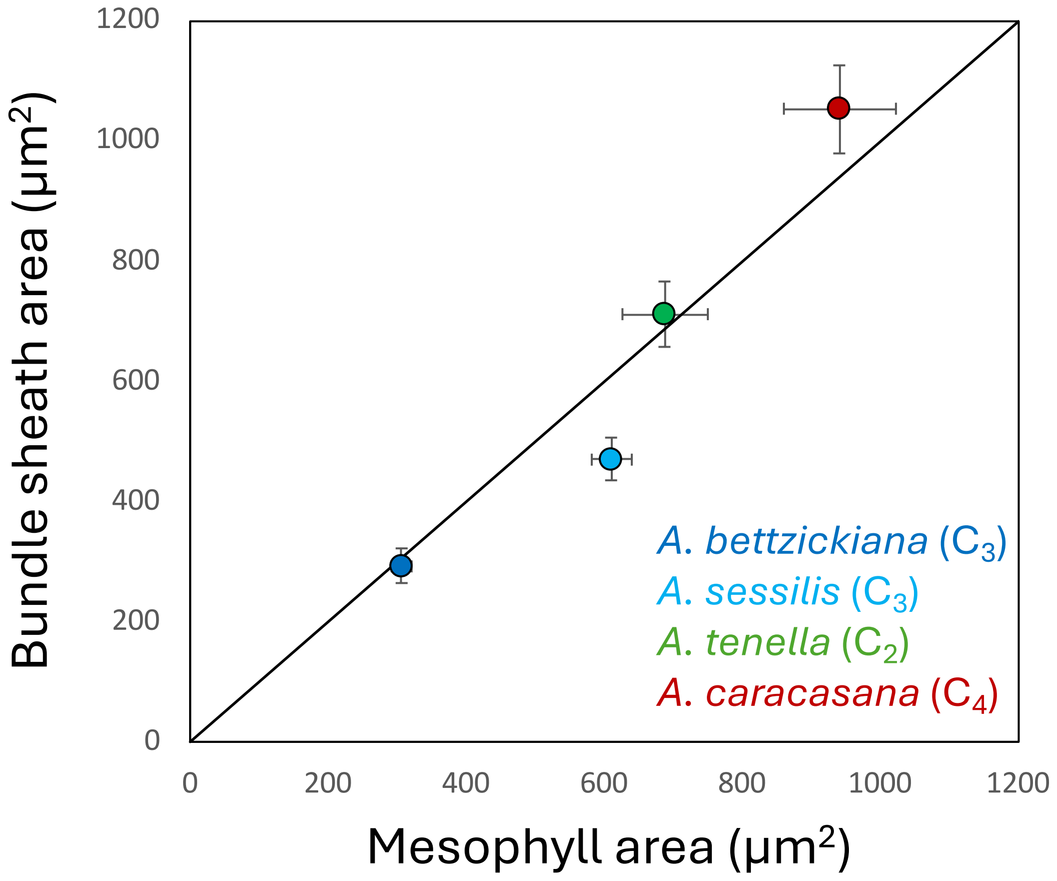

Figure S5. Comparison of bundle sheath and mesophyll tissue areas. Mean bundle sheath and mesophyll tissue areas (± SE) measured on transmission electron microscope images are plotted against each other in C_3 (blues; *A. bettzickiana* and *A. sessilis*), C_2 (green; *A. tenella*), and C_4 (red; *A. caracasana*). We were unable to obtain mesophyll area data for *A. pungens*. These data show that both bundle sheath and mesophyll tissue area increases from C_3 to C_2 and C_4 species. A 1:1 line is plotted in black to highlight the relative ratios of bundle sheath to mesophyll tissue. Points above the line (*i.e.*, C_2 and C_4) have more bundle sheath than mesophyll tissue, whilst points on the line (*A. bettzickiana*, C_3) have similar and points below the line (*A. sessilis*, C_3) have less bundle sheath compared to mesophyll tissue.
